# Refraktivchirurgische Versorgung eines Keratokonuspatienten mittels kombinierter Excimerlaserablation und Hornhautquervernetzung (Athen-Protokoll)

**DOI:** 10.1007/s00347-020-01312-1

**Published:** 2021-01-08

**Authors:** B. Mudarisov, S. J. Linke, J. Steinberg

**Affiliations:** 1 Augenklinik, Universitätsklinikum Hamburg-Eppendorf, Martinistr. 52, 20251 Hamburg, Deutschland; 2 Augenarztpraxis zentrumsehstärke, Martinistr. 64, 20251 Hamburg, Deutschland

## Anamnese

Ein männlicher 33-jähriger Patient stellt sich aufgrund einer progredienten Visusverschlechterung am linken Auge bei progressivem Keratokonus in der refraktiven Sprechstunde des zentrumsehstärke, der Augenarztpraxis auf dem Gelände des Universitätsklinikums Hamburg-Eppendorf, vor. Anamnestisch bestand „früher“ eine deutlich bessere Sehleistung auf dem linken Auge, die Führerscheinsehtest vor einigen Jahren wurde ohne Anmerkungen bestanden.

## Befund

**Figure 1:**
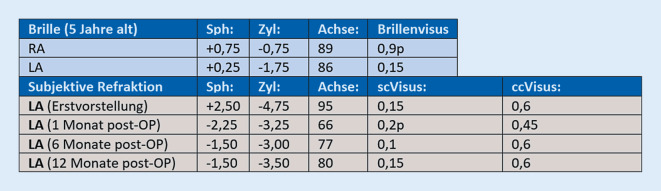

**Figure 2:**
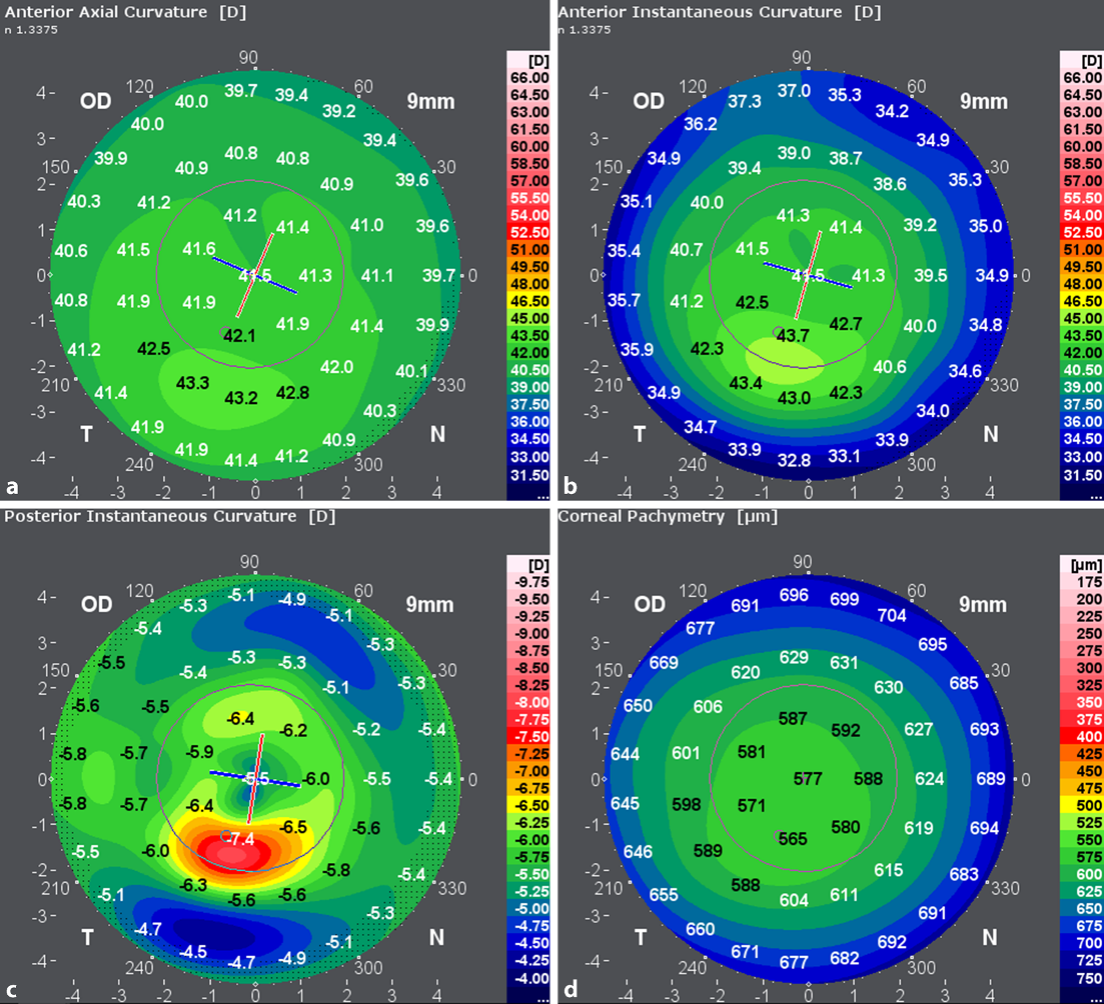

Die zum Zeitpunkt der Erstvorstellung erhobenen Refraktionswerte sind in Abb. 1 dargestellt. Topo- und tomographische Vermessung der Hornhaut mittels Galilei G6 (Ziemer Ophthalmic Systems AG, Port, Switzerland) in Abb. 2 und 3.

**Figure 3:**
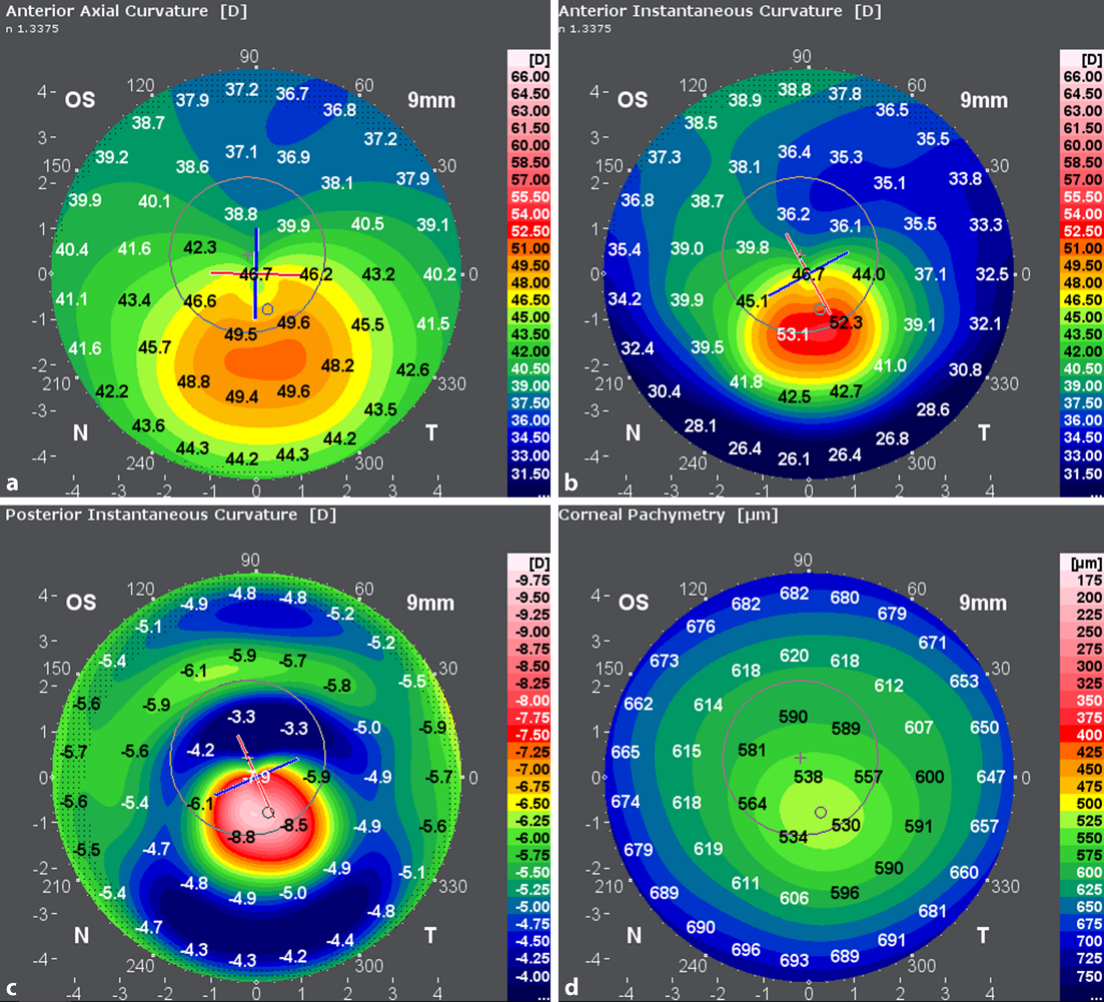

## Diagnose

L > R klinisch manifester Keratokonus.

## Therapie und Verlauf

Eine Korrektur mittels Brillengläser bei gleichzeitig bestehender Kontaktlinsenunverträglichkeit war für den Patienten subjektiv unzureichend. Basierend auf der Anamnese und den messbaren Refraktionsveränderungen (Abb. 1) wurde am linken Auge eine Progression diagnostiziert. Da zudem ein deutlicher Wunsch des Patienten nach einer „wenn möglich“ verbesserten Sehleistung bestand, wurde eine Kombinationstherapie mittels „corneal collagen cross-linking“ (CXL) und photorefraktiver Keratektomie (PRK) entsprechend dem Athen-Protokoll als Therapieoption besprochen [1]. Aufgrund der zentral flachen Hornhautkrümmung und refraktiven Hyperopie wurde eine ebenfalls mögliche Implantation eines MyoRinges primär nicht empfohlen [2].

**Figure 4:**
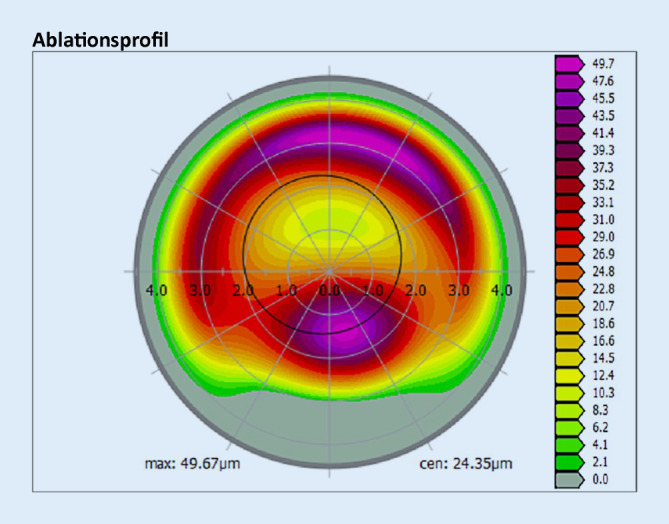

Nach erfolgter Aufklärung erhielt der Patient am linken Auge eine Therapie entsprechend dem Athen-Protokoll. Hierbei wurde, basierend auf den topographischen Daten der Hornhaut, ein Ablationsprofil für den Excimerlaser erstellt, welches als Ziel eine maximal mögliche Regularisierung der Hornhautoberfläche hat (Abb. 4). Der maximale punktuelle Abtrag wurde entsprechend dem Protokolls auf 50 µm limitiert. Direkt im Anschluss an die Excimerlaserablation wurde eine Hornhautquervernetzung zur Stabilisierung der Hornhaut durchgeführt. Die Behandlung verlief komplikationslos und regelrecht. Postoperativ erhielt der Patient Ofloxacin 3 mg/ml, Diclofenac-Natrium 1 mg/ml sowie Tränenersatzmittel (TEM), die zunächst stündlich, im Verlauf mit reduzierter Frequenz getropft werden sollen. Nach der Entfernung der Kontaktlinse (KL) wurde auf Dexamethasondihydrogenphosphat-Dinatrium-Augentropfen (AT) 1,3 mg/ml über insgesamt 50 Tage mit abnehmender Applikationsfrequenz (und weiterhin TEM) umgestellt. Geplante postoperative Kontrollen fanden am Tag 1, 5 (KL-Entfernung) sowie nach 1, 3, 6, 9 und 12 Monaten statt. Aufgrund der langsamen Visusverbesserung wurden auch zwischenzeitlich auf Wunsch des Patienten zusätzliche Kontrollen durchgeführt. Der Verlauf der postoperativen Refraktionswerte und der Visusentwicklung (unkorrigierter Visus – scVisus – UDVA, bestkorrigierter Visus – ccVisus – BCVA) sind in Abb. 1 dargestellt.

**Figure 5:**
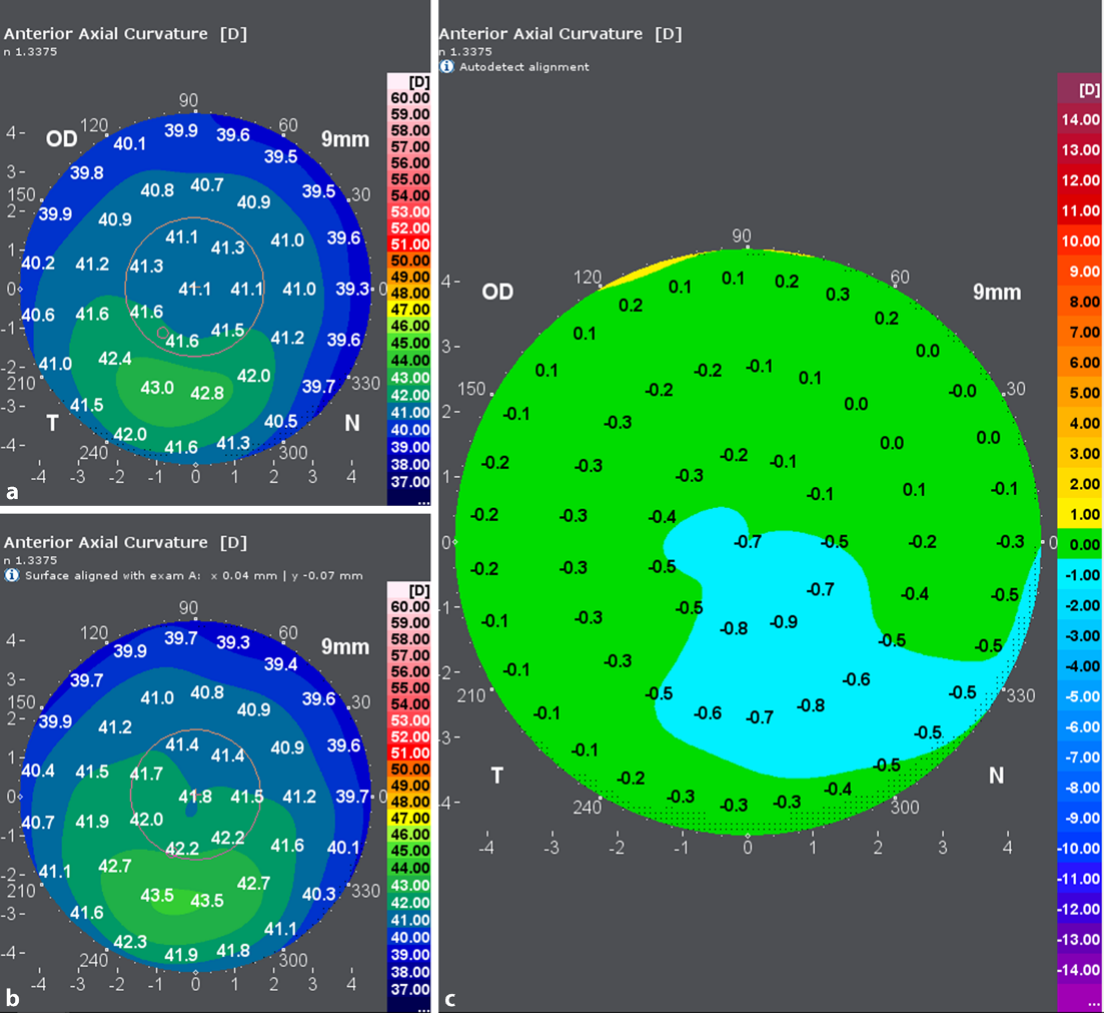

Bei den Verlaufskontrollen in den ersten Monaten nach Operation zeigten sich sehr gute Ergebnisse hinsichtlich der Morphologie der Hornhaut mit einer fast vollständigen Aufklarung des stromalen Gewebes (Haze Fantes +1). Hinsichtlich der Refraktion zeigte sich eine deutliche Myopisierung bei gleichzeitiger Reduktion des Astigmatismus. Es konnte trotz Ausschluss einer weiteren Keratokonusprogression und einer deutlich regularisierten Hornhauttopographie (Abb. 5 und 6) kein Visusanstieg gegenüber dem Ausgangsvisus nachgewiesen werden. Eine Prüfung mittels stenopäischer Lücke ergab ebenfalls einen maximalen Visus von 0,6. Im Rahmen der 6‑Monats-Kontrolle zeigte sich ein auf dem Ausgangsniveau von 0,6 stabilisierter bestkorrigierter Visus (BCVA). Bei dem vorerst letzten Kontrolltermin 12 Monate post-OP konnten weiterhin morphologisch wie funktionell stabile Befunde erhoben werden. Der BCVA blieb seit der letzten Kontrolle unverändert. In der Kontrastvisustestung mit und ohne Blendung (Mesotest) wurde die bestmögliche Kontraststufe von 1:2 selbst ohne Brillengläser erreicht. Die Spektralmikroskopie der kornealen Endothelzellen im Rahmen der letzten Vorstellung ergab eine reguläre Zelldichte (CD) am behandelten Auge von 2810 Zellen/mm^2^. Vergleichsmessungen der Hornhauttopographie beider Augen vor- und 12 Monate nach der Behandlung sind in Abb. 5 und 6 dargestellt. Das rechte Auge stellte sich im Rahmen der Verlaufskontrollen morphologisch und funktionell unverändert dar.

**Figure 6:**
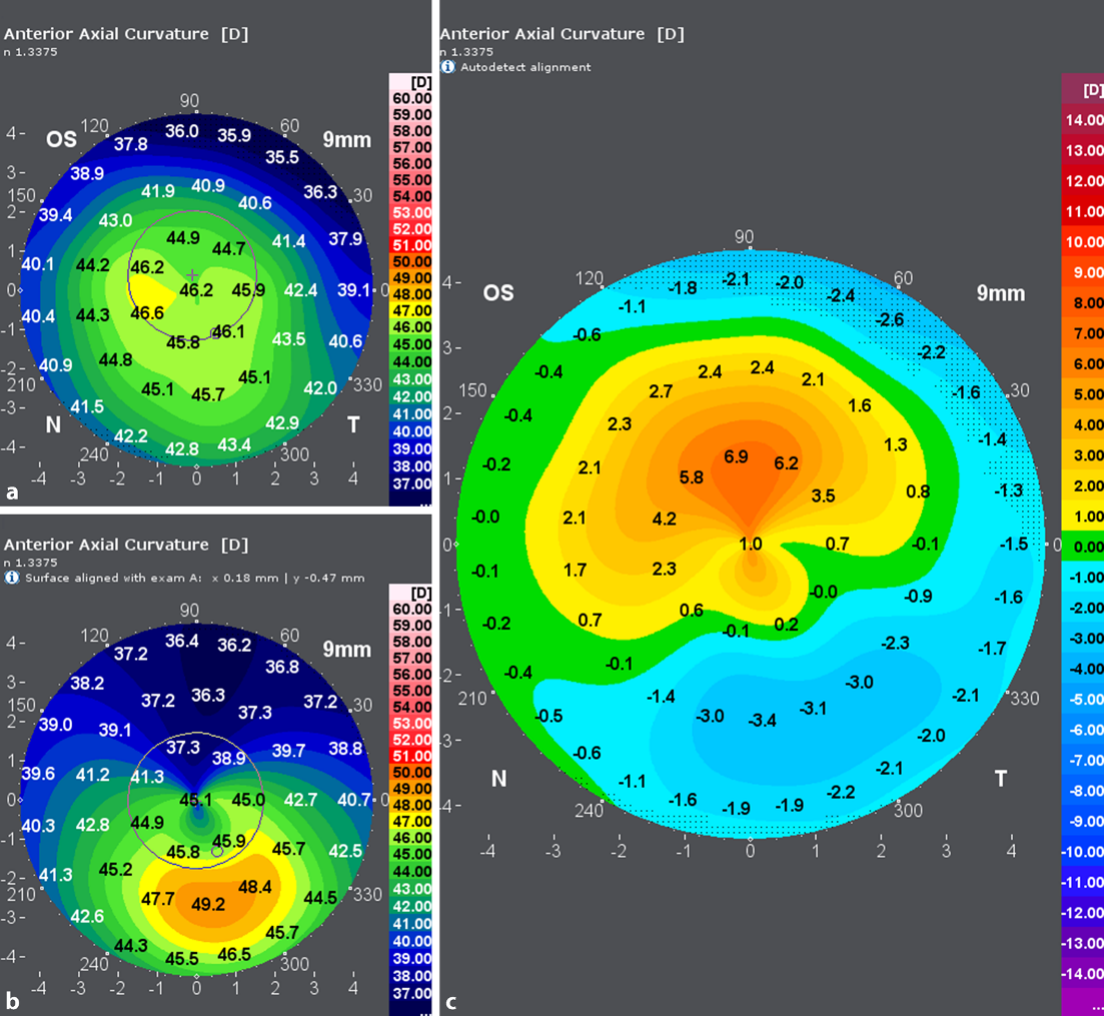

## Diskussion

Mittels moderner Topographie- und Tomographieverfahren lassen sich auch frühe subklinische Formen des Keratokonus mit einer höheren Sensitivität und Spezifität diagnostizieren [3]. Rein refraktiv wirksame Therapieoptionen umfassen Korrekturen durch Hilfsmittel wie Brillengläser, formstabile Kontaktlinsen und Implantation von Ringsegmenten (ICRS). Dies sind aber rein symptomatische Behandlungen ohne biomechanisch stabilisierenden/progressionsstoppenden Einfluss auf die Grunderkrankung. Je nach vorliegendem Ausgangsvisus kommen bei progressiver Erkrankung eine Hornhautquervernetzung (CXL), eine Kombination von Excimerlaserabtrag + CXL (z. B. Athen-Protokoll), Ringsegmentimplantation + CXL oder aber auch die Implantation von kompletten Kunststoff(PMMA)-Ringen in die Hornhaut (MyoRing, Dioptex) infrage [1, 3–6]. Seit Kurzem existieren zudem neue Therapieansätze mittels Bowman-Schicht-„inlay“- und -„onlay“-Transplantation sowie die Implantation von allogenen Gewebsringsegmenten (CAIRS) [7].

Der Ansatz des Athen-Protokolls, entwickelt von Kanellopoulos et al., ist zum einen eine Verbesserung der refraktiven Eigenschaften der Hornhaut durch den gezielten, die Oberfläche regularisierenden Gewebsabtrag mittels des Excimerlasers (PRK), zum anderen durch ein unmittelbar anschließendes CXL eine Stabilisierung des verbleibenden stromalen Hornhautgewebes [8]. In Langzeitkontrollen von über 10 Jahren konnten sowohl eine anhaltende Stabilisierung der Hornhautmorphologie als auch eine signifikante Zunahme der Sehleistung nachgewiesen werden [1].

In unserem Fall wurde, um einen zentral möglichst starken regularisierenden Effekt zu erzielen, nicht nur im Konusbereich Gewebe abgetragen, sondern auch im Bereich der peripheren Hornhaut. Der periphere Abtrag abseits des Konus führt indirekt zu einer Aufsteilung des Hornhautgewebes zentral des peripheren Abtrags. Dieses Prinzip wird auch im Rahmen einer hyperopen refraktiven Laserbehandlung (z. B. LASIK) genutzt. Nachteil dieser partiell zusätzlichen zentralen Aufsteilung ist eine zunehmende Myopisierung des Patienten mit dem Risiko einer (ggf. ungewollt hohen) Anisometropie. Diese könnte in leichten Fällen mit Brillengläsern, alternativ mittels Kontaktlinsen ausgeglichen werden.

Im Vergleich zur Literatur zeigte sich in unserem Fall leider keine messbare Verbesserung des Visus [1, 8]. Die maximale Ablationstiefe betrug 50 µm. Trübungen des Hornhautgewebes konnten nach abgeschlossener Heilung wenige Monate nach Operation sicher ausgeschlossen werden.

Eine weitere mögliche Erklärung für den fehlenden BCVA-Anstieg wäre das Vorhandensein postoperativer Endothelschäden [9]. Auch dies lag in unserem Fall nicht vor.

Die wahrscheinlichste Erklärung für den ausbleibenden Visusanstieg in unserem Fall (und auch allgemein bei Keratokonuspatienten stets zu bedenken) ist das nicht auszuschließende Vorliegen einer (zumindest leichten) Amblyopie. Dies würde auch erklären, warum der Patient selbst mit stenopäischer Lücke nicht über den BCVA von 0,6 hinauskam. Leider wurde präoperativ eine stenopäische Visusprüfung aufgrund der subjektiv deutlichen Verschlechterung des Visus bei gemessenen cc Visus von 0,6, der Angabe des Bestehens des Führerscheinsehtests „ohne Probleme“ und der guten Erklärbarkeit der Visusreduktion durch die stark irreguläre Hornhaut nicht durchgeführt.

Nach zwischenzeitlichem Kontaktlinsenanpassungsversuch, der zu keiner nennenswerten subjektiven Visusverbesserung führte (was auch für die Theorie der leichten Amblyopie sprechen würde), ist der Patient nun mittels Brillengläser versorgt und subjektiv mit seiner Sehleistung zufrieden.

Der Keratokonus bleibt nach wie vor eine herausfordernde Erkrankung. Dank der voranschreitenden Entwicklung und der hohen Präzision bildgebender Verfahren lässt sich die Erkrankung heutzutage in vielen Fällen früh diagnostizieren [10]. Wissenschaftliche Erfahrungen im Bereich der kombinierten Behandlung mittels CXL und PRK sowie Forschungsdaten im Bereich weiterer Therapiemöglichkeiten geben dem Hornhautchirurgen eine zunehmende Auswahl an Behandlungsmethoden und ermöglichen gleichzeitig eine deutliche Individualisierung der Therapie [1, 3, 7, 8]. Da sich der Krankheitsverlauf vor und nach der Behandlung jedoch sehr unterschiedlich darstellen kann, sind stets hohe Aufmerksamkeit und regelmäßige Kontrollen der Patienten erforderlich.

Wichtig ist zu beachten, dass eine alleinige Excimerlaserablation bei Keratokonuspatienten eine absolute Kontraindikation darstellt. Zudem sollte bedacht werden, dass die Studiendaten von Kanellopoulos et al. zwar stabile Befunde auch 10 Jahre nach Behandlung mittels Excimerablation + CXL angeben [1], jedoch gibt es klinisch Hinweise auf ein ggf. Nachlassen des Crosslinking Effektes nach vielen Jahren aufgrund des natürlichen „Gewebsumbaus“ (quervernetztes stromales Gewebe wird durch nicht-quervernetztes ersetzt; Studiendaten hierzu stehen noch aus). Dem gegenüber steht die natürliche Entwicklung der Hornhaut (Zugewinn an Rigidität), welche dazu führt, dass mit dem Älterwerden der Patienten die Progressionswahrscheinlichkeit auf natürlichem Wege nachlässt [11]. Schlussfolgernd ist daraus abzuleiten, dass Patienten mit Keratokonus unabhängig von ihrer Behandlung über viele Jahre nachverfolgt werden sollten.
